# Development and Validation of Artificial Intelligence Models for Prognosis Prediction of Juvenile Myoclonic Epilepsy with Clinical and Radiological Features

**DOI:** 10.3390/jcm13175080

**Published:** 2024-08-27

**Authors:** Kyung Min Kim, Bo Kyu Choi, Woo-Seok Ha, Soomi Cho, Min Kyung Chu, Kyoung Heo, Won-Joo Kim

**Affiliations:** 1 Department of Neurology, Severance Hospital, Yonsei University College of Medicine, Seoul 03722, Republic of Korea; 2 Department of Biomedical Systems Informatics, Yonsei University College of Medicine, Seoul 03722, Republic of Korea; 3 Department of Neurology, Gangnam Severance Hospital, Yonsei University College of Medicine, Seoul 03722, Republic of Korea

**Keywords:** juvenile myoclonic epilepsy, prognosis, machine learning, artificial intelligence, magnetic resonance imaging

## Abstract

**Background:** Juvenile myoclonic epilepsy (JME) is a common adolescent epilepsy characterized by myoclonic, generalized tonic–clonic, and sometimes absence seizures. Prognosis varies, with many patients experiencing relapse despite pharmacological treatment. Recent advances in imaging and artificial intelligence suggest that combining microstructural brain changes with traditional clinical variables can enhance potential prognostic biomarkers identification. **Methods**: A retrospective study was conducted on patients with JME at the Severance Hospital, analyzing clinical variables and magnetic resonance imaging (MRI) data. Machine learning models were developed to predict prognosis using clinical and radiological features. **Results**: The study utilized six machine learning models, with the XGBoost model demonstrating the highest predictive accuracy (AUROC 0.700). Combining clinical and MRI data outperformed models using either type of data alone. The key features identified through a Shapley additive explanation analysis included the volumes of the left cerebellum white matter, right thalamus, and left globus pallidus. **Conclusions:** This study demonstrated that integrating clinical and radiological data enhances the predictive accuracy of JME prognosis. Combining these neuroanatomical features with clinical variables provided a robust prediction of JME prognosis, highlighting the importance of integrating multimodal data for accurate prognosis.

## 1. Introduction

Juvenile myoclonic epilepsy (JME) is a prevalent adolescent epilepsy, accounting for approximately 10% of all epilepsies [1,2]. Characterized by recurrent myoclonic seizures, primarily in the shoulders and limbs, JME typically begins during puberty and is often accompanied by generalized onset motor tonic–clonic seizures and generalized onset non-motor (absence) seizures [3,4]. Classified under idiopathic generalized epilepsy (IGE), the prognosis for JME with pharmacological treatment is relatively favorable, with approximately 60% of the patients achieving five years or more of seizure freedom on medication, and approximately 25% maintaining remission without medication [5,6]. However, many patients relapse after stopping medication, necessitating lifelong treatment. Meta-analyses suggest that approximately 35% of the patients exhibit drug-refractory epilepsy [7,8].

Clinical factors influencing the prognosis with antiseizure medication include female gender, a younger age of onset, a history of absence seizures, praxis-induced seizures, childhood absence epilepsy, comorbid psychiatric disorders, family history, epileptiform asymmetries on Electroencephalogram (EEG), and the absence of photoparoxysmal response [9,10]. While brain magnetic resonance imaging (MRI) in patients with JME appears visually normal, recent advancements in quantitative and functional MRI analysis reveal differences in structural and functional connectivity compared to healthy controls, along with widespread neocortical thinning as the disease progresses [11,12].

Despite the progress in imaging techniques, studies analyzing quantitative imaging features for prognosis in JME remain scarce. Recent efforts have utilized machine learning and deep learning to develop diagnostic and prognostic models for various neurological diseases, including epilepsy [13,14]. Some studies have combined clinical information and brain MRI images to predict drug response, but these models often underperform due to the insufficient integration of comprehensive variables [15,16].

To address this gap, our study develops and validates artificial intelligence models that combine clinical and imaging variables to predict the prognosis of drug treatment response in JME.

## 2. Materials and Methods

### 2.1. Data and Participants

This study was approved by the institutional review board (no. 4-2021-1196), and the requirement for informed consent was waived. A retrospective review of consecutive patients who presented with seizures and visited the epilepsy clinic of the Severance Hospital (a tertiary care center) between January 2000 and May 2021 was conducted [17]. The patients diagnosed with JME by epilepsy specialists were included. The exclusion criteria were as follows: (1) patients with less than a 3-year follow-up, and (2) participants who underwent a 2D protocol MRI. 

The initial diagnosis of JME was confirmed by reviewing the medical records of the institution’s neurologists. The diagnosis was based on the clinical and EEG features set by the International League against Epilepsy. The patients with JME experienced both generalized onset motor myoclonic and generalized onset motor tonic–clonic seizures. Additionally, the EEG findings consistently demonstrated generalized polyspikes or spike-and-wave complexes in all the patients with JME. The MRI readings were confirmed to be normal by board-certified neuroradiologists. 

**Figure 1:**
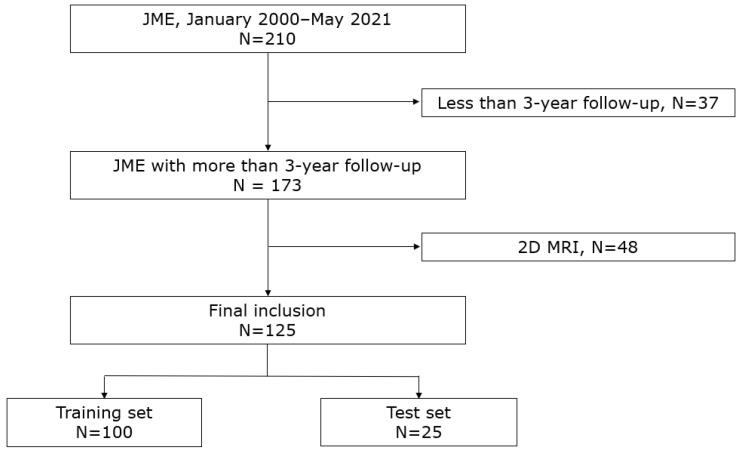
Flow chart depicting the study subjects.

The clinical variables collected from the patients included age, sex, age at onset, the duration of disease, treatment history of seizures, number of antiseizure medications attempted prior to the visit, a family history of epilepsy, a history of febrile seizures, and a history of absence seizures. The patients who were seizure-free for 2 years or longer at any time after diagnosis were considered to have a favorable outcome. We randomly divided the entire subjects into training and test sets in an 8:2 ratio and used them for training and testing the artificial intelligence model. The study flow is shown in Figure 1. 

### 2.2. Image Acquisition and Processing

All the MRI scans were performed using a 3T MRI system with an eight-channel sensitivity-encoding head coil (Achieva or Ingenia, Philips Healthcare; TrioTim, Siemens). The 3D T1-weighted images with isotropic voxels were obtained using a spoiled gradient echo sequence. For preprocessing, the T1-weighted images were resampled to a uniform spatial resolution of 1 × 1 × 1 mm^3^, followed by the N4 bias field correction and z-score normalization of the images. All the images were submitted to imaging experts for visual analysis. Structural MRI data were processed using the FreeSurfer 6.0.0 software. The processing pipeline included several key steps: Raw Digital Imaging and Communications in Medicine (DICOM) files were converted to Neuroimaging Informatics Technology Initiative (NIfTI) format and imported into the FreeSurfer environment. The automated segmentation of the brain was performed using the recon-all pipeline, which included motion correction, skull stripping, and the segmentation of cortical and subcortical regions [18,19,20]. The resulting segmentations were visually inspected for accuracy, and manual corrections were applied as necessary. Following segmentation, the volumetric measurements of various brain regions were obtained from the automatically generated aseg.stats file. Key 29 regions of interest (ROI), including bilateral thalamus, bilateral caudate, bilateral putamen, bilateral globus pallidus, bilateral hippocampus, bilateral amygdala, bilateral nucleus accumbens, bilateral ventral diencephalon, bilateral choroid plexus, bilateral cerebellum (cortex and white matter), optic chiasm, brain stem, and corpus callosum (anterior, mid-anterior, central, mid-posterior, and posterior), were extracted and compiled into a comprehensive dataset for the subsequent statistical analysis. This process ensured the consistent and accurate measurement of brain region volumes across all the subjects. 

### 2.3. Machine Learning Models

Machine learning models were developed using a dataset composed of clinical variables from the patients with JME and volume data from the various regions of interest (ROI). The dataset was divided into training and test sets to train and evaluate the models.

We employed six machine learning algorithms (decision tree, random forest, XGBoost, LightGBM, support vector machine (SVM), and artificial neural network (ANN)) to predict post-surgery delirium. Each model was trained on the training set, with hyperparameters tuned using grid search with cross-validation on the training data. The models’ performances were evaluated on the testing set based on accuracy and the area under the receiver operating characteristic curve (AUROC).

A decision tree classifier was implemented using the scikit-learn library, with tree depth restricted to prevent overfitting, and the maximum depth set through cross-validation. The random forest model, also implemented in scikit-learn, consisted of an ensemble of decision trees, with parameters such as the number of trees (n_estimators) and the maximum depth of each tree (max_depth) optimized via cross-validation. XGBoost was used to train a gradient boosting model that minimizes a regularized objective function, with hyperparameters including n_estimators, max_depth, learning_rate, and subsample rates tuned to optimize performance. LightGBM, another gradient boosting framework, was chosen for its efficiency with large datasets, with parameters such as num_leaves, max_depth, learning_rate, and n_estimators tuned to find the optimal settings. The SVM model classified the data by finding the optimal hyperplane that maximizes the margin between classes. A kernel function was applied to handle non-linear relationships in the data. An ANN model with input, hidden, and output layers was used. Neurons applied weighted sums and non-linear activation functions, and the model was trained using backpropagation and gradient descent to minimize the loss function.

Shapley additive explanations (SHAP) were adopted to verify the explainability of the AI models [21,22]. These values highlight the key factors in predicting JME prognosis, providing insights into model patterns.

## 3. Results

### 3.1. Clinical Characteristics

The clinical variables are summarized in Table 1. Among the 125 patients, 85 were seizure-free for over 2 years (favorable prognosis), whereas 40 were not (poor prognosis). In the favorable prognosis group, the proportion of males was higher compared with the poor prognosis group (60.0% vs. 40.0%, *p* = 0.036). There were no significant differences between the two groups regarding the age, onset age, and epilepsy duration. Similarly, there were no differences in the family history, febrile seizure history, or the presence of absence seizures between the groups. Regarding treatment, there were no differences in the proportion of treated patients, the number of antiseizure medications used, or the use of valproic acid, lamotrigine, levetiracetam, and topiramate. Additionally, the follow-up duration was comparable between the two groups.

### 3.2. Radiological Characteristics 

The radiological characteristics are summarized in Table 2. Comparing the volume data of the regions of interest (ROI) between the two groups demonstrated that the volumes of the left amygdala (1739.9 ± 263.4 vs. 1601.7 ± 358.2, *p* = 0.017) and the right hippocampus (4396.6 ± 417.7 vs. 4128.8 ± 825.7, *p* = 0.017) were significantly smaller in the poor prognosis group. No significant differences were observed between the groups in the volumes of the other bilateral subcortical structures, the brain stem, the corpus callosum, or the total intracranial volume.

### 3.3. Performances of Machine Learning Models

Table 3 shows the performance of the six machine learning models—logistic regression, random forest, XGBoost, LightGBM, SVM, and ANN—developed using the training set and validated on an independent test set. Among these, XGBoost achieved the highest performance (AUROC, 0.700), followed by LightGBM (AUROC, 0.618), random forest (AUROC, 0.7317), SVM (AUROC, 0.500), logistic regression (AUROC, 0.431), and ANN (AUROC, 0.425).

**Figure 2:**
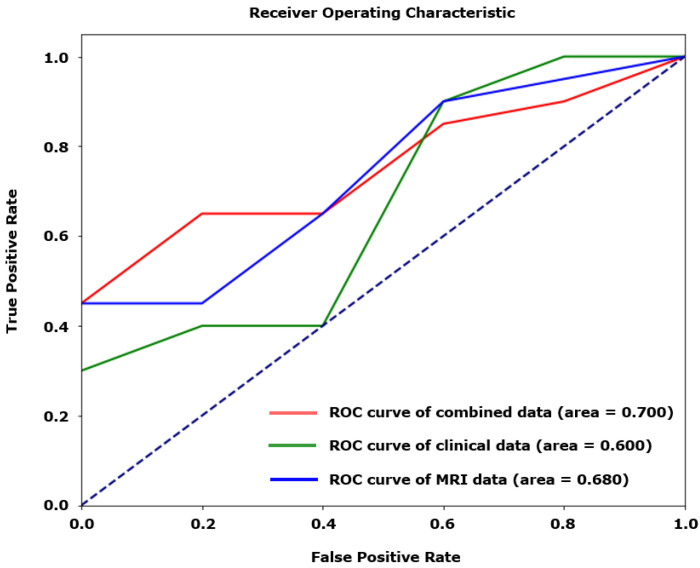
Receiver operating characteristic (ROC) curves for models using clinical, radiological, and combined variables to predict poor prognosis of JME.

When the best-performing XGBoost model was developed using only clinical data, AUROC was 0.0.600. Using only the MRI data, AUROC was 0.680. Combining both the clinical and MRI data improved AUROC to 0.700 (Figure 2).

### 3.4. Feature Importances

**Figure 3:**
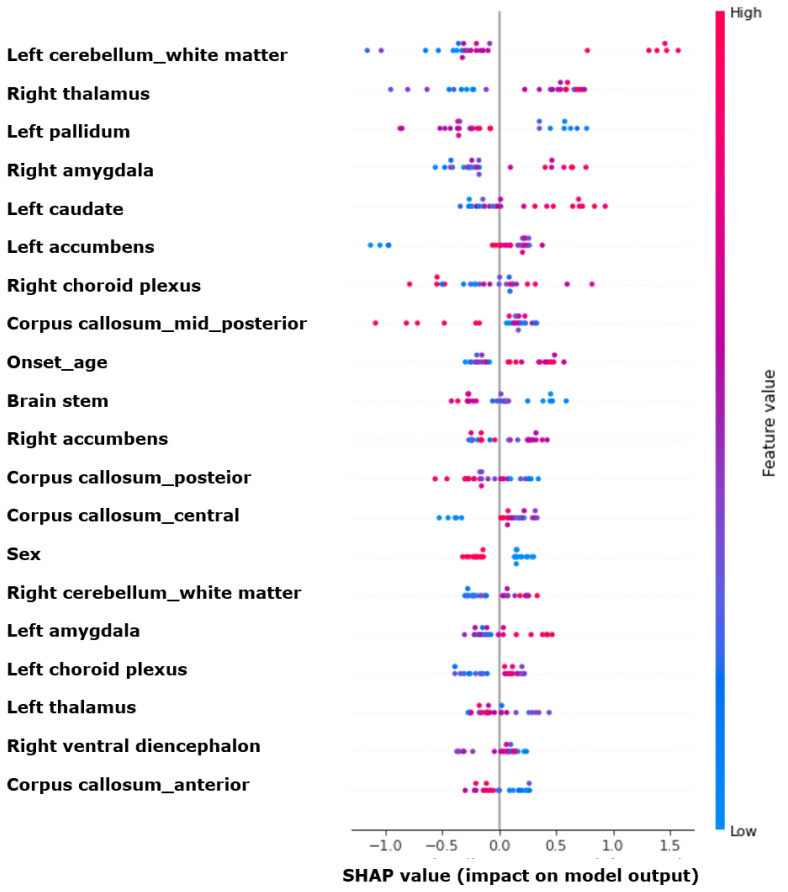
SHAP value summary plot for the XGBoost model. SHAP, Shapley additive explanations; XGBoost, extreme gradient boosting.

Figure 3 illustrates the feature importance using SHAP values. Among the top ten features, the five most significant MRI variables were as follows: left cerebellum white matter, right thalamus, left globus pallidus, right amygdala, and left caudate. Following these are the left nucleus accumbens, right choroid plexus, corpus callosum mid-posterior, onset age, and brain stem. 

## 4. Discussion

The key findings of this study are as follows:

1. The volumes of the left amygdala and right hippocampus, along with male gender, were associated with prognosis;The XGBoost model demonstrated a performance of approximately 0.700 in predicting prognosis, with higher performance observed when combining the clinical and radiological variables;
2. The cerebellum, thalamus, and globus pallidus were crucial for the machine learning model’s prediction of prognosis.

In our clinical variables, male gender was associated with a favorable prognosis, consistent with previous studies on the prognosis of JME [6,23]. This may be due to the broader range of antiseizure medications available to males compared to females of childbearing age. Additionally, this study identified the amygdala and hippocampus as the structures associated with prognosis. These structures have shown differences in comparisons between the patients with JME and healthy controls, and are involved in emotion and cognition, which may influence prognosis in JME.

This study develops a novel prognostic prediction model for JME based on clinical and radiological variables. Recent efforts have concentrated on developing various AI models that utilize clinical variables for diagnosis and treatment in clinical settings [16,24]. Our research group’s previous studies have investigated the use of radiological variables in diagnosing and classifying JME and other generalized epilepsies. [25]. These studies demonstrated that microstructural changes in the brain MRI of patients with JME could enhance diagnosis and classification. This study further shows that combining clinical and radiological variables yields superior predictive performance compared to using either type of variable alone. Thus, it is crucial to use multimodal data in AI models to enhance prognostic prediction capabilities. It is expected that this model will assist physicians, especially those who are not epileptologists, in diagnosing and explaining the prognosis of epilepsy patients based on clinical and MRI data. Increasing the generalizability of these models through diverse datasets is essential for improving the application of AI in epilepsy treatment.

In our study, the SHAP value analysis of the most efficient XGBoost model revealed that the cerebellum and thalamus were the two most important variables for predicting prognosis. While cerebellar volume reduction has been associated with the use of antiseizure medications (ASMs) such as phenytoin, none of our patients were treated with phenytoin. Additionally, there were no significant differences between the groups in terms of the number of ASMs used or the duration of epilepsy [26]. Previous studies have demonstrated that patients with JME show a reduced cerebellar white matter volume compared to healthy controls [27,28]. This finding aligns with the theory that an altered cerebello-thalamo-cortical network contributes to JME pathogenesis, potentially influencing treatment response and prognosis. Moreover, the thalamus undergoes microstructural changes in JME, with volume alterations in specific regions being well documented [29,30]. A previous study has also suggested the potential involvement of altered thalamo-frontal connectivity with the widespread network in seizure regulation [31,32]. In our study, the thalamus was similarly identified as a crucial feature in determining prognosis, reinforcing its importance in the disease’s underlying mechanisms and clinical outcomes.

This study has several limitations. First, being a single-center study, it has a small sample size, and external validation will be crucial in future research to verify reproducibility. Second, the multimodal approach was limited, utilizing only the volume analysis of T1 3D MRI among the various radiological variables, and EEG signals were not used in the model development. Third, the study included a heterogeneous sample with varied treatment exposures and disease durations. Future research with larger samples and stricter inclusion criteria may provide more robust results. 

This study has several notable strengths. First, it presents the novel prognosis of JME by combining clinical and radiological variables, demonstrating that radiological variables significantly enhance the prognostic prediction capability alongside clinical variables. We identified models with appropriate performance levels using various machine-learning techniques. Second, the study confirmed that both clinical and radiological variables, previously identified as relevant to JME in earlier studies, showed differences according to prognosis. The importance of these variables in the machine learning models was also related to the mechanisms of JME, providing clinical explainability.

## 5. Conclusions

This study demonstrated the clinical utility of a machine learning model combining clinical and radiological variables to predict the prognosis of JME. It highlighted the potential of AI-supported care using explainable machine learning models in the clinical management of epilepsy.

## Figures and Tables

**Table 1:** Demographic and clinical characteristics of the patients in the study.

	Favorable Prognosis (*n* = 85)	Poor Prognosis (*n* = 40)	*p*-Value
Age (years)	23.5 ± 8.7	23.2 ± 7.7	0.843
Male sex, *n* (%)	51 (60.0)	16 (40.0)	0.036
Onset age (years)	15.2 ± 4.5	15.2 ± 4.8	0.991
Epilepsy duration (years)	8.3 ± 9.4	8.0 ± 8.2	0.888
Family history, *n* (%)	16 (18.8)	4 (10.0)	0.209
Febrile seizure history *, *n* (%)	9 (10.6)	5 (12.5)	0.767
Absence seizure, *n* (%)	31 (36.5)	14 (35.0)	0.873
Treated, *n* (%)	44 (51.8)	24 (60.0)	0.388
Number of ASMs	2 (1–3)	2 (1–3)	0.577
VPA, *n* (%)	65 (76.5)	30 (75.0)	0.857
LTG, *n* (%)	39 (45.9)	21 (52.5)	0.490
LEV, *n* (%)	40 (47.1)	24 (60.0)	0.177
TPM, *n* (%)	17 (20.0)	6 (15.0)	0.501
Follow-up duration (years)	13.9 ± 6.7	11.1 ± 7.2	0.178

* Fisher’s exact test was used. ASM, antiseizure medication; VPA, valproic acid; LTG, lamotrigine; LEV, levetiracetam; TPM, topiramate.

**Table 2:** Radiologic features and their association with the prognosis of JME.

	Favorable Prognosis (*n* = 85)	Poor Prognosis (*n* = 40)	*p*-Value
Left			
Thalamus	8054.0 ± 844.4	7748.2 ± 1393.7	0.131
Caudate	3569.1 ± 433.8	3458.2 ± 643.6	0.259
Putamen	5075.5 ± 619.2	4902.2 ± 895.1	0.211
Pallidum	2073.8 ± 244.5	2017.6 ± 353.9	0.303
Hippocampus	4180.6 ± 415.2	4027.1 ± 630.2	0.107
Amygdala	1739.9 ± 263.4	1601.7 ± 358.2	0.017
Nucleus accumbens	511.4 ± 98.5	286.6 ± 133.8	0.246
Ventral diencephalon	4175.7 ± 457.7	4003.7 ± 621.1	0.084
Choroid plexus	437.0 ± 163.7	427.5 ± 159.2	0.765
Cerebellum–cortex	56,595.1 ± 5734.4	54,113.8 ± 8452.0	0.057
Cerebellum–white matter	14,800.9 ± 1846.9	14,133.2 ± 2293.1	0.084
Right			
Thalamus	7574.4 ± 775.1	7219.0 ± 1275.0	0.056
Caudate	3637.9 ± 444.1	3544.3 ± 567.1	0.318
Putamen	5130.4 ± 617.7	4985.8 ± 809.3	0.273
Pallidum	1977.6 ± 238.8	1942.0 ± 284.8	0.467
Hippocampus	4396.6 ± 417.7	4128.8 ± 825.7	0.017
Amygdala	1848.7 ± 279.3	1744.6 ± 361.0	0.080
Nucleus accumbens	577.2 ± 105.1	554.5 ± 117.2	0.280
Ventral diencephalon	4173.3 ± 444.1	4017.0 ± 562.6	0.095
Choroid plexus	430.6 ± 154.9	422.9 ± 188.5	0.809
Cerebellum–cortex	56,266.6 ± 5838.5	53,800.4 ± 8392.4	0.059
Cerebellum–white matter	14,242.4 ± 2001.1	13,540.7 ± 2200.4	0.079
Midline			
Brainstem	21,182.7 ± 2266.9	20,565.8 ± 3631.8	0.248
Optic-chiasm	154.3 ± 58.7	140.1 ± 60.8	0.213
Corpus callosum			
Anterior	862.7 ± 141.4	836.8 ± 158.5	0.360
Mid-anterior	669.4 ± 181.2	666.1 ± 171.8	0.922
Central	688.8 ± 172.1	660.2 ± 171.5	0.388
Mid-posterior	552.8 ± 103.9	560.6 ± 128.1	0.720
Posterior	990.4 ± 178.0	982.0 ± 205.2	0.815
Total intracranial volume	1,581,418.5 ± 175,060.8	1,502,151.6 ± 250,957.1	0.077

The presented values represent the volume of each brain region, with the units in mm^3^.

**Table 3:** Performances of machine learning models on the test set.

Models	Accuracy	Precision	Recall	F1-Score	AUROC
Logistic Regression	0.600	0.560	0.600	0.565	0.431
Random Forest	0.680	0.664	0.680	0.652	0.580
XGBoost	0.680	0.816	0.680	0.712	0.700
Light GBM	0.560	0.486	0.560	0.505	0.618
SVM	0.640	0.410	0.640	0.500	0.500
ANN	0.600	0.400	0.600	0.480	0.425

AUROC, area under the receiver operating characteristic curve; XGBoost, extreme gradient boosting; LightGBM, light gradient boosting machine; SVM, support vector machine; ANN, artificial neural network.

## Data Availability

Anonymized data relevant to this study will be shared upon request with a qualified investigator pending appropriate Institutional Review Board approvals.
